# Genome-wide identification of myeloblastosis gene family and its response to cadmium stress in *Ipomoea aquatica*

**DOI:** 10.3389/fpls.2022.979988

**Published:** 2022-08-23

**Authors:** Zheng Liu, Yuxin Zhang, Muhammad Ahsan Altaf, Yuanyuan Hao, Guangzhen Zhou, Xinyu Li, Jie Zhu, Wuqiang Ma, Zhiwei Wang, Wenlong Bao

**Affiliations:** ^1^Key Laboratory for Quality Regulation of Tropical Horticultural Crops of Hainan Province, College of Horticulture, Hainan University, Haikou, China; ^2^College of Tropical Crops, Hainan University, Haikou, China; ^3^Hainan Yazhou Bay Seed Laboratory, Sanya Nanfan Research Institute of Hainan University, Sanya, China

**Keywords:** *cis*-acting element, gene duplication mode, gene expression, heavy metal stress, transcription factor

## Abstract

The myeloblastosis (MYB) proteins perform key functions in mediating cadmium (Cd) tolerance of plants. *Ipomoea aquatica* has strong adaptability to Cd Stress, while the roles of the *I. aquatica MYB* gene family with respect to Cd stress are still unclear. Here, we identified a total of 183 *MYB* genes in the *I. aquatica* genome (*laMYB*), which were classified into 66 1R-type *IaMYB*, 112 2R-type *IaMYB*, four 3R-type *IaMYB*, and one 4R-type *IaMYB* based on the number of the MYB repeat in each gene. The analysis of phylogenetic tree indicated that most of *IaMYB* genes are associated with the diverse biological processes including defense, development and metabolism. Analysis of sequence features showed that the *IaMYB* genes within identical subfamily have the similar patterns of the motif distributions and gene structures. Analysis of gene duplication events revealed that the dispersed duplication (DSD) and whole-genome duplication (WGD) modes play vital roles in the expansion of the *IaMYB* gene family. Expression profiling manifests that approximately 20% of *IaMYB* genes had significant role in the roots of *I. aquatica* under Cd stress. Promoter profiling implied that the differentially expressed genes might be induced by environmental factors or inherent hormones and thereby execute their function in Cd response. Remarkably, the 2R-type *IaMYB157* with abundant light-responsive element G-box and ABA-responsive element ABRE in its promoter region exhibited very strong response to Cd stress. Taken together, our findings provide an important candidate *IaMYB* gene for further deciphering the molecular regulatory mechanism in plant with respect to Cd stress.

## Introduction

The increasing discharge of heavy metals into the ecosystems has created global environmental problems with the intensification of industrial and agricultural activities. Cadmium (Cd) is an ubiquitous heavy metal contaminant, which poses serious threats to the biosphere due to its long biological half-life, non-biodegradability, water solubility, and high mobility (Sarwar et al., 2010; Cirmi et al., 2021; Kumar et al., 2021). Cd restrains the normal functioning of plants by perturbing the physiological function of plasma membrane, reducing the bioactivity of enzymes, and causing the burst of intracellular reactive oxygen species (ROS) (Janicka-Russak et al., 2008; Singh et al., 2018; Guo et al., 2022). To better adapt to the Cd-contaminated circumstances, plants struggling with Cd have evolved sophisticated regulatory networks to mitigate Cd-induced deleterious effects.

Transcription factors (TFs) act as crucial regulators for plant Cd accumulation and tolerance, which execute their functions by switching “on” or “off” downstream gene expression. Several TFs that involved in plant response to Cd stress have been reported, including basic helix–loop–helix protein (bHLH) (Xu et al., 2017), dehydration-responsive element-binding protein (DREB) (Akbudak et al., 2018), metal-responsive element-binding transcription factor (MTF) (Sun et al., 2015), ethylene-responsive factor (ERF) (Tian et al., 2022) basic leucine Zipper (bZIP) (Lu et al., 2022), WRKY (Sheng et al., 2019), and MYB (Sapara et al., 2019).

Myeloblastosis (MYB) proteins comprise a superfamily of TFs in a broad range of species and perform versatile functions in diverse biological processes. As reported, the first member of the *MYB* gene family, an oncogene *v-MYB*, was discovered from the avian myeloblastosis virus more than 40 years ago (Lipsick and Wang, 1999). Subsequently, the proto-oncogene *c-MYB*, a cellular homolog of *v-MYB* gene, was identified (Lipsick, 2010). In 1987, the first *MYB* gene in plants was found in *Zea mays* (Paz-Ares et al., 1987). Since then, an increasing number of *MYB* genes in diverse plant species have been widely identified and studied. Structurally, MYB proteins are composed of a highly conserved DNA-binding domain (DBD) that is located at the N-terminus and a variable transcriptional regulation domain (TRD) that is located at the C-terminus. The imperfect repeat (R) sequences of 50–55 amino acid residues are a core component of the DBD within MYB proteins. Each R structure has evolutionarily conserved tryptophan residues (W) and interval sequences which was consisted of 18–19 variable amino acids, forming a helix-turn-helix (HTH) motif to participate in DNA binding. Based on the similarity to the Rs in c-MYB, the Rs of MYB in the plant were designated as R1, R2, and R3, respectively (Saikumar et al., 1990; Dubos et al., 2010). According to the arrangement of the Rs, MYB TFs in the plant can be subdivided into four distinct types, i.e., 1R-type MYB (R1/2/3-MYB), 2R-type MYB (R2R3-MYB), 3R-type MYB (R1R2R3-MYB), and 4R-type MYB (R1R2R2R1/2-MYB). Remarkably, the 2R-type *MYB* genes are specific to plants and are the richest *MYB* in most plant species, which underwent a rapid expansion during the evolutionary process and thereby greatly facilitated the evolution of *MYB* genes in plant realms (Ambawat et al., 2013; Jiang and Rao, 2020). Several studies demonstrated that MYB TFs perform key functions in plant tolerance to Cd stress. For instance, Cd-induced *AtMYB49* can significantly increase Cd accumulation in *Arabidopsis thaliana* by directly or indirectly regulating the expression of heavy metal-associated isoprenylated plant proteins (HIPPs) and ABA-inhibited metal transporter iron-regulated transporter 1 (IRT1), which is also involved in a feedback mechanism to modulate Cd uptake in plants by interacting with the basic region/Leu zipper TF abscisic acid-insensitive 5 (ABI5; Zhang et al., 2019). Genome-wide identification of *AtMYB* genes and its expression profiling showed that approximately 20% of *AtMYB* genes were engaged in Cd response. The transcript abundance of a *Boehmeria nivea BnMYB2* was highly enhanced by Cd stress. Further study confirmed that the overexpression of *35S:BnMYB2* can impart Cd tolerance to *A. thaliana* (Zhu et al., 2020). In general, these studies have deepened our knowledge to understand the molecular regulatory function of *MYBs* in plant response to Cd stress.

*Ipomoea aquatica* is one of the only two edible plants in the family Convolvulaceae, which is widely distributed in tropical and subtropical areas (Hao et al., 2021). The characteristic of the high capacity of Cd accumulation together with strong adaptability to Cd stress brought *I. aquatica* into focus for studying plant response to Cd stress (Wang et al., 2007; Shen et al., 2017). However, little is known about the *MYB* gene family in *I. aquatica* and its expression patterns in response to Cd stress due to a lack of genomic information. In our previous work, we constructed a high-quality chromosome-level genome assembly of *I. aquatica* (Hao et al., 2021), which provides excellent sequence resources for comprehensive analysis of the *MYB* gene family in *I. aquatica*. In the present study, we performed genome-wide identification of *IaMYB* genes by conducting the bioinformatic search of *I*. *aquatica* genome and analyzed their sequence features, gene duplication modes, selective pressure, expression patterns under Cd stress, and *cis*-acting elements. Remarkably, 2R-type *IaMYB157* with abundant light-responsive element G-box and ABA-responsive element ABRE in its promoter region exhibited very strong response to Cd stress. Our findings provide an important candidate *IaMYB* gene for further deciphering the molecular regulatory mechanism in plant with respect to Cd stress.

## Materials and methods

### Identification of *MYB* genes in *Ipomoea aquatica* (*IaMYBs*)

A high-quality chromosome-level genome database of *I. aquatica* (BioProject: PRJCA002216) that we constructed in our previous work was used for the identification of *IaMYBs*. The *MYB* genes in *A. thaliana* (*AtMYBs*) were retrieved from TAIR^1^ and used for the phylogeny analysis in this study. The hidden Markov model (HMM) profile of the MYB protein domain (PF00249) was downloaded from the Pfam database.^2^ The *IaMYB* candidates were preliminarily identified using HMM search (*E*-value < 10^−5^) according to the previously published methods (Krogh et al., 2001). Subsequently, the putative *IaMYBs* were further confirmed by using the NCBI CD-search,^3^ SMART,^4^ and Pfam^5^ database.

### Analysis of physicochemical parameters of IaMYB proteins

The ExPASy^6^ and TBtools were performed to analyze the physicochemical parameters of each IaMYB proteins, including the number of amino acids, molecular weight (MW), theoretical isoelectric point (pI), instability index, aliphatic index and grand average of hydropathicity (GRAVY). In addition, the PIant-mPLoc online program^7^ was employed to predict the subcellular localization of each IaMYB protein.

### Analysis of phylogenetic relationships of *MYB* genes in *Ipomoea aquatica* and *Arabidopsis thaliana*

Multiple sequence alignments were carried out using ClustalW with default parameters, and then a neighbor-joining (NJ) method within MEGA-X (Kumar et al., 2018) was conducted to construct a phylogenetic tree. The reliability of the phylogenetic tree was tested by 1,000 bootstrap replications. The Evolview^8^ program was performed to further manipulate and annotate the phylogenetic tree (He et al., 2016).

### Analysis of conserved motifs, gene structures, and chromosome distribution of *IaMYBs*

The MEME online tool^9^ was performed to analyze conserved motifs shared among *IaMYB* genes. The parameters of MEME were set as follows: the site distribution: Zero or one occurrence per sequence; the number of motifs = 10; the width of motifs = 50–53 residues. The WebLogo program^10^ was used to envision the sequence logo of *R*2 and *R*3 domains distributed in 2R-type IaMYB proteins. The TBtools software was employed to map exon-intron structures of *IaMYBs* based on CDS of each *IaMYB* gene and its corresponding genomic DNA sequences (Chen et al., 2020). Furthermore, The TBtools software was performed to visualize the information of genome-wide chromosomal density and the distribution of *IaMYBs* across all chromosomes of *I. aquatica* based on the genome annotation files. The genes distributed on scaffolds were excluded.

### Analysis of syntenic relationships, gene duplication events, and Ka/Ks of *MYBs*

Syntenic relationships of *MYB* genes in interspecies (*I*. *aquatica* vs. *Ipomoea batatas*, *I. aquatica* vs. *A*. *thaliana*, and *I. aquatica* vs. *Oryza sativa*) and intraspecies were analyzed using TBtools software with default parameters. Gene duplication events of IaMYB duplicated gene pairs were detected by performing the DupGen_finder pipeline, which includes whole-genome duplication (WGD), tandem duplication (TD), proximal duplication (PD), transposed duplication (TRD), and dispersed duplication (DSD; Qiao et al., 2019). In addition, the Nei-Gojobori method within TBtools was used to calculate the non-synonymous substitution rates (Ka) and the synonymous substitution rates (Ks) of *IaMYBs*.

### Analysis of *cis*-acting regulatory elements

Upstream sequences (2,000 bp) from the start codon of CDS of *IaMYB* genes were considered as promoter regions, which were extracted from the genome database of *I. aquatica*. Afterward, the *cis*-acting elements of those sequences were detected using the PlantCARE program.^11^

### Sampling of plant materials

Seeds of *I. aquatica* were soaked in 55°C water for 20 min, and washed with tap water 3 times. Subsequently, the seeds were put on the blotting paper and moistened with tap water for germination. Germinated seeds were planted in Hoagland nutrient solution and cultivated for 25 days under the growth conditions of 16 h light (31°C) and 8 h darkness (26°C) with the replacement of fresh nutrient solution by every 3 days. The 25-day seedlings were divided into two groups: Control group (CK), the seedlings were cultivated in Hoagland nutrient solution; Cd-treated group, the seedlings were cultivated in Hoagland nutrient solution supplemented with CdCl_2_·2H_2_O to the final concentration of 5 mg/l Cd^2+^. The roots of seedlings from the control group and Cd-treated group were collected after 4-day treatment, which was immediately frozen in liquid nitrogen and stored at −80°C until use. Each treatment was independently replicated three times.

### Transcriptome analysis of *IaMYB* genes

Based on the RNA-seq data (BioProject: PRJNA812778), the transcriptome analyses were carried out to evaluate the expression patterns of *IaMYB* genes under Cd treatment. In this section, we quantified the transcript abundances of *IaMYB* genes by calculating the value of FPKM (fragments per kilobase per million mapped reads) of each gene, and defined the differentially expressed genes (DEGs) by the criteria as follows: |log2FC| > 1, FDR < 0.05, and *p*-value < 0.05. The TBtools software was used to generate the gene expression heatmap based on the FPKM value of each gene.

### Quantitative real-time PCR (qRT-PCR) analysis of randomly selected *IaMYBs*

Total RNA was extracted from the roots of seedlings in the CK and Cd-treated group by using RNAprep pure Plant Kit (Tiangen, China) according to the manufacturer’s protocol. The concentration, quality, and integrity of total RNA were evaluated on a NanoDrop 2000 (ThermoFisher, United states) and checked by using gel electrophoresis. The high-quality total RNA was used as a template to synthesize cDNA using the Reverse Transcription Kit (Tiangen, China). The specific primers used for quantitative real-time PCR (qRT-PCR) were designed by using Primer5 software (Supplementary Table S9). The total reaction volume of qRT-PCR was 20 μl, containing 10 μl of 2 × SYBR Green Master Mix (TaKaRa, China), 0.5 μl cDNA template, 0.5 μl of forward and reverse primers, and 8.5 μl PCR-grade water. The final results were analyzed by using the 2^−ΔΔCt^ method. The *GAPDH* gene encoding the glyceraldehyde-3-phosphate dehydrogenase was used as an internal housekeeping gene. GraphPad Prism 8 was used to produce the final figures. Each experiment was independently replicated three times.

## Results

### Identification and characterization of *IaMYB* genes

**Figure 1:**
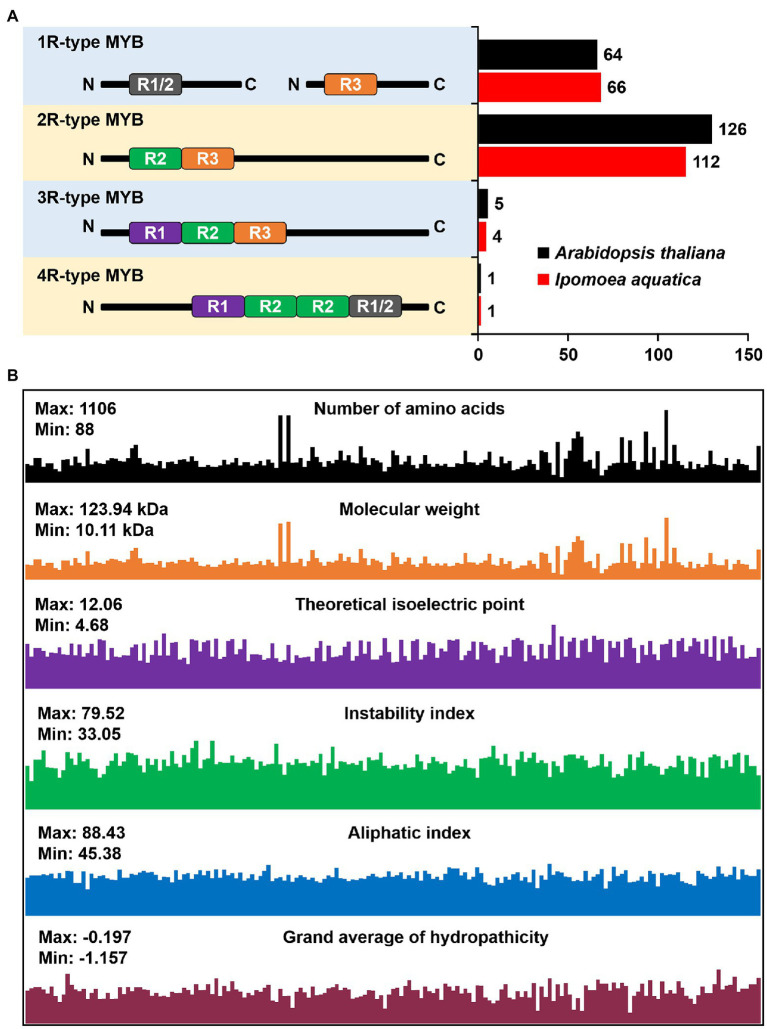
Identification of *IaMYB* genes. **(A)** The number of *MYB* gene in *Ipomoea aquatica* and *Arabidopsis thaliana*. The *MYB* genes were classified into four types accordding to the arrangement of *MYB* repeat in each gene, namely 1R-type *MYB*, 2R-type *MYB*, 3R-type *MYB*, and 4R-type *MYB* (Dubos et al., 2010). **(B)** The physicochemical properties of IaMYB proteins.

To identify the member of IaMYB TFs, we used PF00249 as seed sequence to conduct the bioinformatic search of *I. aquatica* genome database. After removing the redundant sequences based on E value and protein structure, a total of 183 *IaMYB* genes were confirmed, which were renamed as *IaMYB1*-*IaMYB183* based on the order of their distribution on chromosomes and scaffolds (Supplementary Table S1). According to the arrangement of imperfect repeats (Rs), *183 IaMYB* genes were classified into distinct four types, namely 1R-type *IaMYB*, 2R-type *IaMYB*, 3R-type *IaMYB*, and 4R-type *IaMYB*, respectively. As shown in Figure 1A, the number of *MYB* gene within each type of *MYB* in *I. aquatica* was consistent with that in *A. thaliana*. The 2R-type *IaMYB* had the largest number of genes with 61.2% (112/183) of the total *IaMYB* genes, while the 4R-type *IaMYB* had the lowest number with only one gene. In addition, the 1R-type *IaMYB* and 3R-type *IaMYB* had 66 and 4 genes, respectively. Besides, as shown in Figure 1B and Supplementary Table S2, the number of amino acids of IaMYB proteins ranged from 88 (IaMYB23) to 1,106 (IaMYB103), and their molecular weight (MW) varied from 10.11 kDa to 123.94 kDa. The value of the theoretical isoelectric point (pI) is a vital parameter for protein purification, which varied from 4.68 (IaMYB56) to 12.06 (IaMYB19). The instability index of 12 out of 183 IaMYB proteins were lower than 40, while the rest were larger than 40. There are 57 of 183 IaMYB TFs have a value of aliphatic index (Ai) greater than 71. IaMYB5 had the highest Ai with 88.43, while IaMYB24 had the lowest Ai with 45.38. The analysis of the grand average of hydropathicity (GRAVY) showed that all IaMYB proteins had negative hydrophobicity. The prediction of subcellular localization showed that all IaMYB proteins localized in the nucleus.

**Figure 2:**
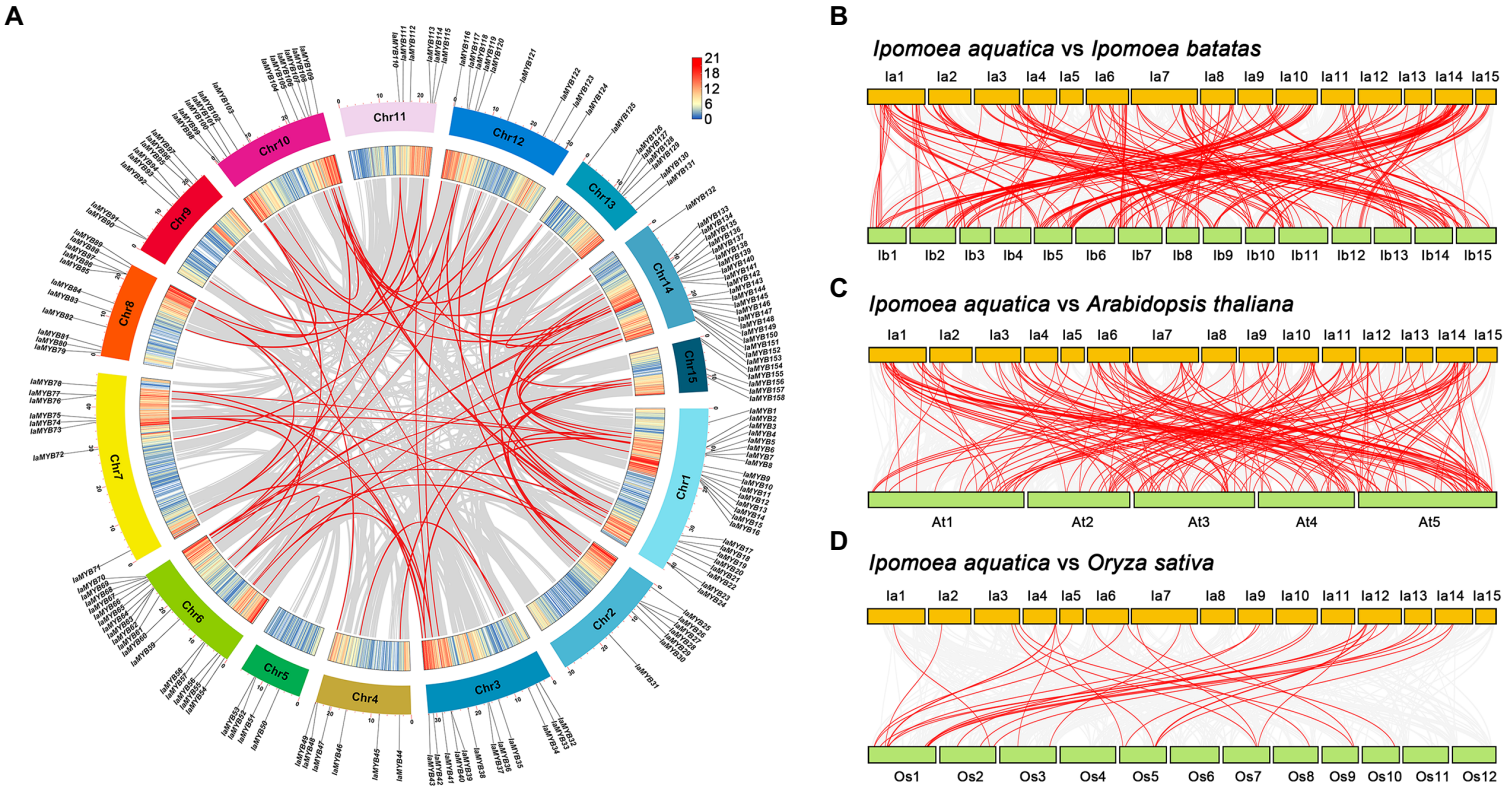
Analysis of *IaMYB* genes at the chromosome level. **(A)** Chromosome distribution of *IaMYB* genes and their collinear duplications in *I. aquatica* genome. The outer circle with different colors show 15 chromosomes of *I. aquatica*; the inner circle shows the gene density of each chromosome. **(B–D)** Synteny analysis of *IaMYB* genes between *I. batatas*, *A. thaliana*, and *O. sativa* genomes, respectively. The gray line linked with each chromosome show all collinear gene pairs between *I. aquatica* and other plant genomes, while the red line show the collinear gene pairs with respect to *IaMYB* genes. Please refer to the Supplementary Table S3 for the detailed information of each gene pairs.

To illustrate the genomic distribution of the *IaMYB* genes, their location on each chromosome were marked based on their physical positions. As shown in Figure 2A, 158 of 183 *IaMYB* genes were mapped across 15 chromosomes (Chr1 to Chr15), while the rest were traced on the scaffolds (Supplementary Table S1). The rank of chromosomes with different number of *IaMYB* genes were as follows: Chr 1 (containing 24 *IaMYBs*) > Chr 14 (containing 22 *IaMYBs*) > Chr 6 (containing 17 *IaMYBs*) > Chr 10 (containing 12 *IaMYBs*) = Chr 3 (containing 12 *IaMYBs*) > Chr 8 (containing 11 *IaMYBs*) > Chr 12 (containing 9 *IaMYBs*) > Chr 7 (containing 8 *IaMYBs*) = Chr 9 (containing 8 *IaMYBs*) > Chr 13 (containing 7 *IaMYBs*) = Chr 2 (containing 7 *IaMYBs*) > Chr 11 (containing 6 *IaMYBs*) = Chr 4 (containing 6 *IaMYBs*) > Chr 15 (containing 5 *IaMYBs*) > Chr 5 (containing 4 *IaMYBs*).

To uncover the distribution of *IaMYB* collinear duplications on each chromosome, synteny analyses were performed. The results showed that a total of 77 *IaMYB* collinear duplications were identified in the genome of *I. aquatica*. Of these, 61 *IaMYB* gene pairs were distributed on almost all chromosomes except for Chr 5, while the rest were spread on the scaffolds (Figure 2A). To provide more insights into the potential evolutionary process of *IaMYB* genes, the synteny relationships of *IaMYB* genes with three representative species were investigated at the whole-genome levels, including dicot *I. batatas* (belongs to the family Convolvulaceae), dicot *A. thaliana* (belongs to the family Brassicaceae), and monocot *O. sativa* (belongs to the family Poaceae) (Supplementary Table S3). The largest number of *IaMYB* homologous gene pairs were detected between *I. aquatica* and *I. batatas* (255; Figure 2B), followed by *I. aquatica* and *A. thaliana* (187; Figure 2C). While, only 39 *IaMYB* homologous gene pairs were found between *I. aquatica* and *O. sativa* (Figure 2D).

### Phylogenetic tree analysis of MYB proteins in *Ipomoea aquatica* and *Arabidopsis thaliana*

**Figure 3:**
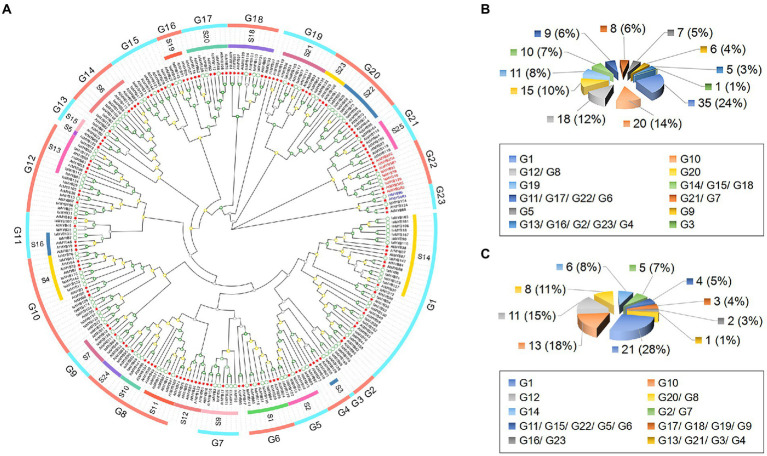
Phylogenetic relationships of MYB proteins in *I. aquatica* and *A. thaliana*. **(A)** The phylogenetic tree contains the 117 IaMYB proteins (including 112 2R-type IaMYB, 4 3R-type IaMYB, and one 4R-type IaMYB) and the 131 AtMYB proteins (including 125 2R-type AtMYB, five 3R-type AtMYBs, and one 4R-type AtMYB). **(B)** The number distribution of MYB proteins in each subgroup. **(C)** The number distribution of IaMYB proteins in each subgroup. The function of the AtMYB proteins in each subgroup were consistent with previous report in *A. thaliana* (Dubos et al., 2010). The green circles represent the IaMYB proteins. The red stars represent the AtMYB proteins. The red letters show the 3R-type MYB in *I. aquatica* and *A. thaliana*. The blue letters show the 4R-type MYB in *I. aquatica* and *A. thaliana*. The green, yellow and gray quadrangle represents the bootstrap value ranged from 80 to 100, 40 to 80, and 0 to 40, respectively.

To predict the possible function of the IaMYB proteins, we constructed a phylogenetic tree using amino acid sequences of MYB proteins in *I. aquatica* and *A. thaliana*. As shown in Figure 3A, the phylogenetic tree contained the 117 IaMYB proteins (including 112 2R-type IaMYBs, four 3R-type IaMYBs, and one 4R-type IaMYB) and the 131 AtMYB proteins (including 125 2R-type AtMYB, five 3R-type AtMYBs, and one 4R-type AtMYB). According to the topology of the phylogenetic tree together with the classification of the AtMYB proteins, 117 IaMYBs were subdivided into 23 subgroups (G1–G23). The total number of MYB protein in different subgroups varied to some extent (Figure 3B). The subgroup G1 had the largest number of IaMYB proteins with 21, while the subgroup G3, G4, G13 and G21 had the lowest number with only one IaMYB proteins (Figure 3C). Except for the IaMYB proteins in the subgroup G2, G3 and G15, all the IaMYB proteins in the different subgroups were well matched with the functional defined AtMYB subgroups that associated with four major function including defense, development, metabolism and differentiation (Supplementary Table S4). For example, the AtMYBs in the subgroup S14 were defined as development-related proteins, which were clustered together with the IaMYBs in subgroup G1. The AtMYBs in the subgroup S2 related to defense responses, which were grouped together with the IaMYBs in the subgroup G5. The AtMYB in the subgroup S7 gathered with the IaMYB in the subgroup G9, which was defined as metabolism that mainly involved in phenylpropanoid pathway and flavanol biosynthesis. The four 3R-type IaMYB proteins (IaMYB39, IaMYB40, IaMYB92, and IaMYB120) and the five 3R-type AtMYB proteins were gathered in the subgroup G22. Intriguingly, the phylogenetic distance between the four 3R-type MYB proteins and the development-associated AtMYB proteins in the subgroup S25 was close. The 4R-type MYB protein from *I. aquatica* and *A. thaliana* were clustered in the same clade within subgroup G23. Besides, the ten AtMYB proteins were not clustered with any IaMYB proteins.

### The sequence features of the R2 and R3 MYB repeat

**Figure 4:**
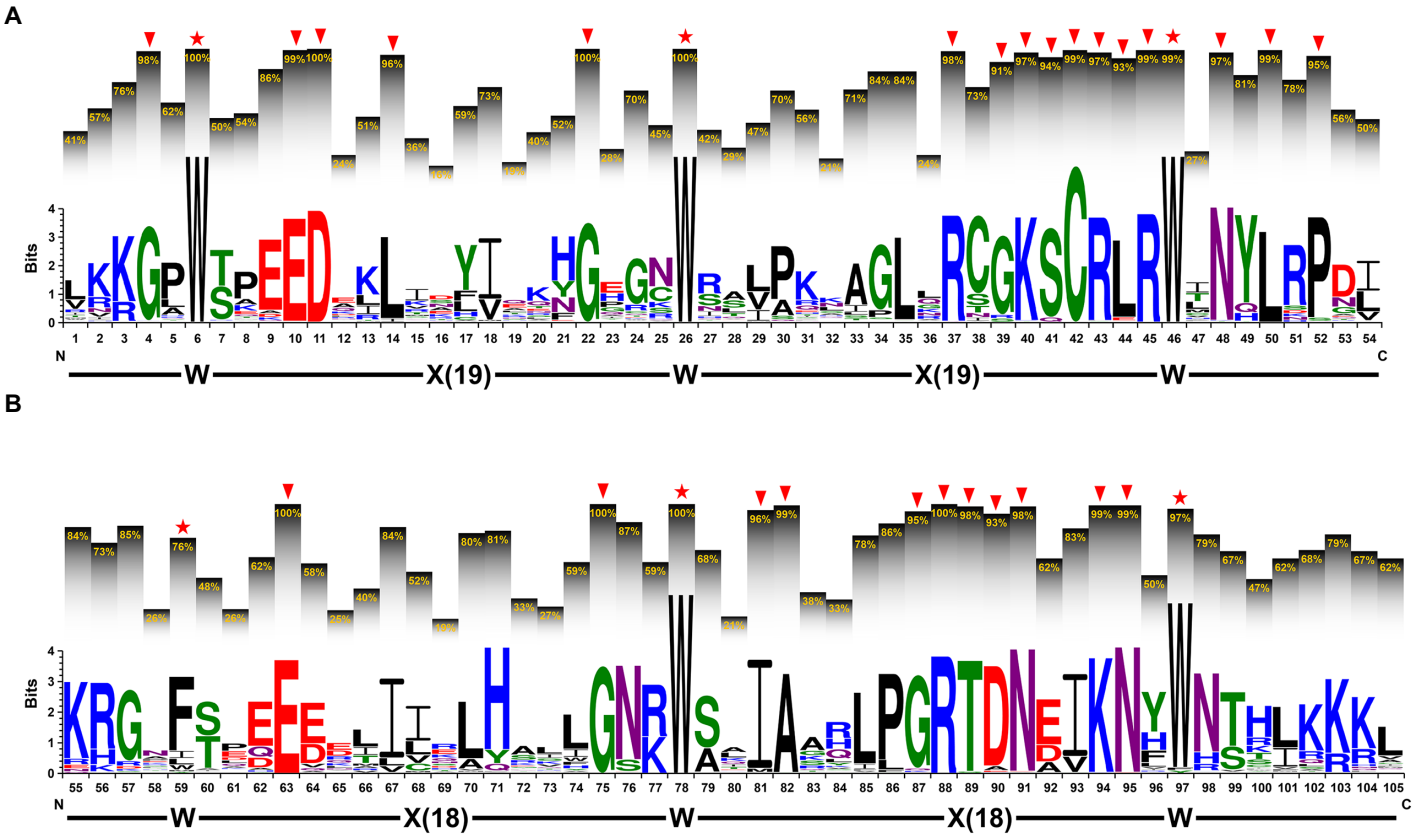
The sequence characteristics of the R2 and R3 MYB repeats. **(A)** The sequence logo of the R2 MYB repeat and **(B)** the R3 MYB repeat. The letters within each stack represent different amino acids and their height indicates the relative frequency of each amino acid at the respective position. The percentage on the histograms show the frequency of the highest conserved amino acid within each stack. The red stars indicate the conserved the highly conserved tryptophan (W) or the relatively conserved phenylalanine (F). The arrows indicate the highly conserved amino acids with frequency greater than 90%.

To clarify the sequence features of the R2 and R3 MYB repeats, we performed multiple sequence alignment using amino acid sequences of 112 2R-type IaMYB proteins. As shown in Figure 4A, the primary structure of the R2 MYB repeat was expressed as [–W–(X19)–W–(X19)–W–], where the W represents the highly conserved tryptophan residues (located on position 6, 26 and 46) and the X19 represents 19 regular interval sequences. In addition, some other highly conserved amino acids with frequency greater than 90% were detected at the different position of the R2 MYB repeat, such as G (position 4, 22 and 39), E (position 10), D (position 11), L (position 14, 44 and 50), R (position 37, 43 and 45), K (position 40), S (position 41), C (position 42), N (position 48), and P (position 52). As shown in Figure 4B, the primary structure of the R3 MYB repeat was expressed as [–F–(X18)–W–(X18)–W–], which contained one relatively conserved phenylalanine (position 54) and two highly conserved W (position 78 and 97) intercalated within 18 regular interval sequences. Furthermore, the results showed that some other amino acids appeared in the R3 MYB repeat with frequency higher than 90%, including E (position 63), G (position 75 and 87), I (position 81), A (position 82), R (position 88), T (position 89), D (position 90), N (position 91 and 95), and K (position 94).

### Conserved motifs, and exon-intron structures of *IaMYB* genes

**Figure 5:**
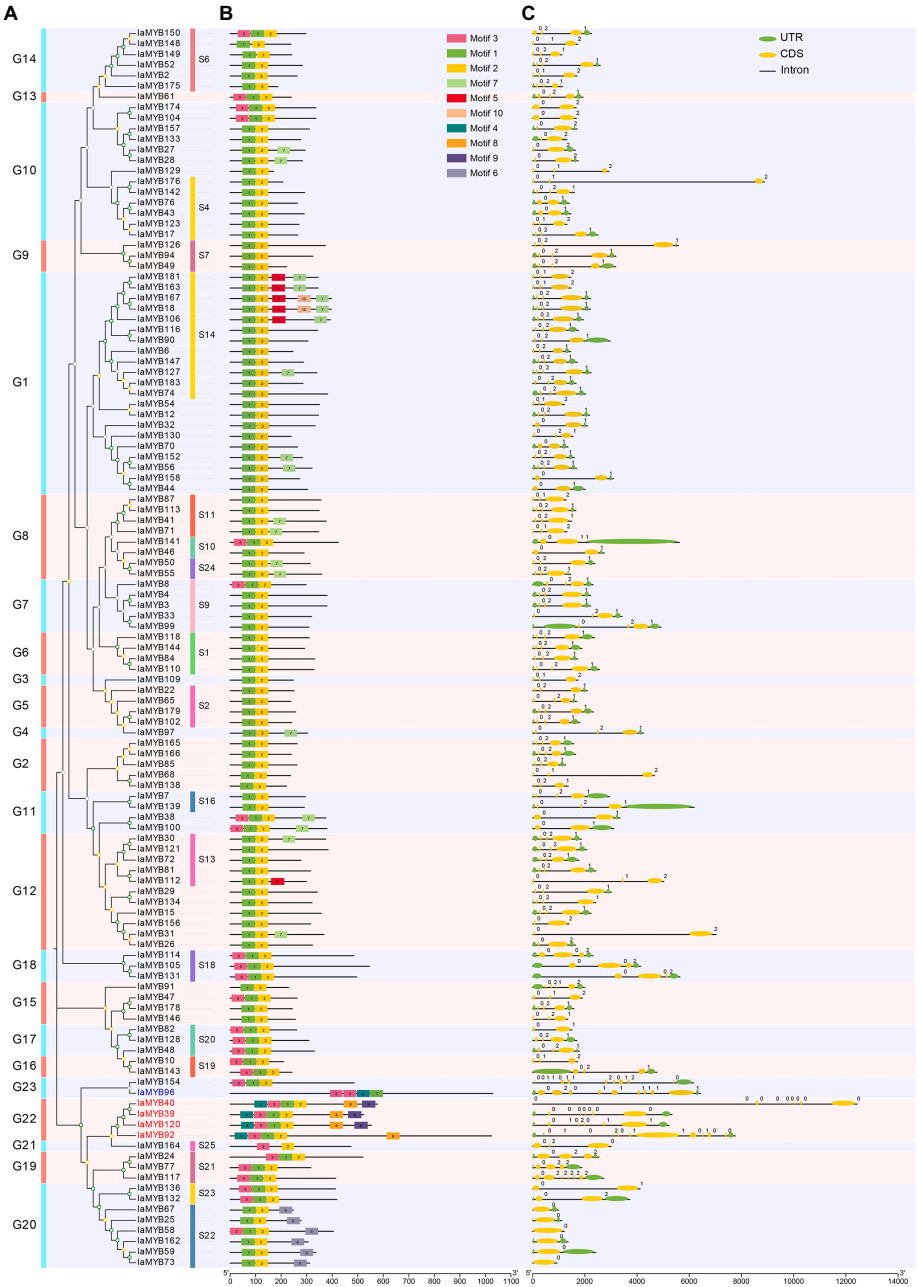
The phylogenetic tree, conserved motif, and exon-intron structures of the *IaMYB* genes. **(A)** The phylogenetic tree of the 117 IaMYB proteins (including 112 2R-type IaMYBs, four 3R-type IaMYBs, and one 4R-type IaMYB). **(B)** The conserved motif of the 117 IaMYB proteins. The rectangles with different colors represent the Motif 1–10; the black solid line represents non-conserved regions; the bar scale shows the length of the amino acids. **(C)** The exon-intron structures of the 117 *IaMYB* genes. The green ellipses represent the exons; the orange ellipses represent an untranslated region (UTR); the black solid lines among the ellipse represent the introns; the bar scale shows the length of the gene.

To investigate the sequence characteristics of the IaMYB TFs, their conserved motifs were analyzed by using MEME program. As a result, a total of ten conserved motifs (Motif 1–10) were detected in 117 IaMYB proteins (including 112 2R-type IaMYBs, four 3R-type IaMYBs, and one 4R-type IaMYBs). As shown in Figure 5A,B, IaMYB proteins in the same subgroup had similar motifs. The Motif 1 and Motif 2 were detected as the most common motif appeared in the IaMYB proteins. The Motif 8 and Motif 9 were specific to 3R-type IaMYBs in the subgroup G22. The Motif 4 only presented in the 3R- and 4R-type IaMYB proteins. The Motif 6 was only detected in the subgroup G20. The analysis of exon-intron structures showed that the number distribution of intron in the 117 IaMYB genes varied from 0 to 11 (Figure 5C and Supplementary Table S1). More than 67% of IaMYB genes had 2 introns and 3 exons. The 3R-type IaMYB92 in the subgroup G22 and the IaMYB154 in the subgroup G23 had the largest number of introns with 11, while the six IaMYB genes in the subgroup G20 (IaMYB25, IaMYB58, IaMYB59, IaMYB67, IaMYB73, and IaMYB162) were not disrupted by an intron, which were clustered with the defense responsive AtMYB proteins in the subgroup S22.

### Analysis of gene duplication events of *IaMYB* genes

**Figure 6:**
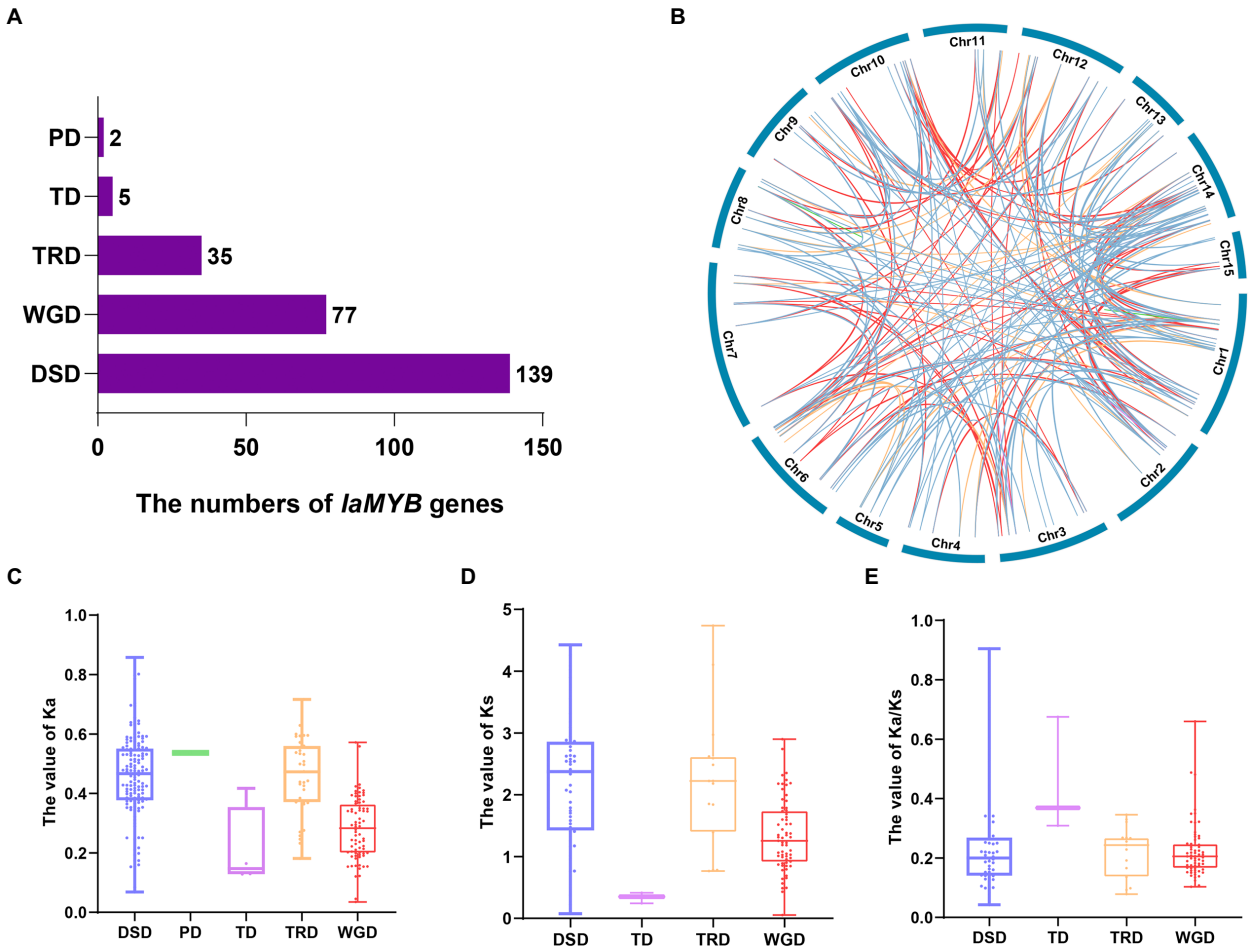
Gene duplication events of *IaMYB* genes. **(A)** The number distribution of the duplicated genes in the five duplication events. **(B)** Chromosome distribution of the duplicated genes in each event. The blue line, green line, purple line, yellow line, and red line linked with each chromosome show the duplicated genes originated from DSD, PD, TD, TRD, and WGD, respectively. **(C–E)** The Ka, Ks, and Ka/Ks distribution of the duplicated genes in the five duplication events.

To understand the primary driven-force in the evolutionary process of *IaMYB* genes, the five gene duplication events (i.e., WGD, TD, PD, TRD, and DSD) were analyzed by the Dup Gen finder pipeline (Supplementary Table S5). As shown in Figure 6A, the number distribution of the duplicated genes in the five duplication modes varied greatly. The DSD mode had the largest number of duplicated genes with 139, followed by the WGD mode with 77. In addition, 35 duplicated genes were found in the TRD mode, and 5 duplicated genes were detected in the TD mode. The PD mode had the lowest number of *IaMYB* duplicated gene pairs with only two. The analysis of chromosome distribution showed that the duplicated genes of each gene duplication mode were unevenly spread on the different chromosomes (Figure 6B).

To disclose the direction of evolution, we calculated the value of Ka (non-synonymous substitution rate), Ks (synonymous substitution rate) and Ka/Ks of *IaMYB* duplicated genes originated from the five duplication events. The results showed that the different modes of duplicated genes presented divergent Ka, Ks, and Ka/Ks distributions (Supplementary Table S5). Overall, the Ka values ranged from 0.035 to 0.86, and the Ks values varied from 0.053 to 4.74. The PD event had the highest median Ka value (0.536), followed by DSD (0.458), TRD (0.456), WGD (0.278), and TD (0.168) (Figure 6C). The rank of the median Ks values were as follows: TRD (2.237) > DSD (2.216) > WGD (1.321) > TD (0.252) (Figure 6D). As shown in Figure 6E, the Ka/Ks ratio of all the *IaMYB* gene pairs were lower than 1, indicating purifying selection played a vital role in the evolutionary process of *IaMYB* gene family.

### Expression patterns of *IaMYB* genes in the root under Cd treatment

**Figure 7:**
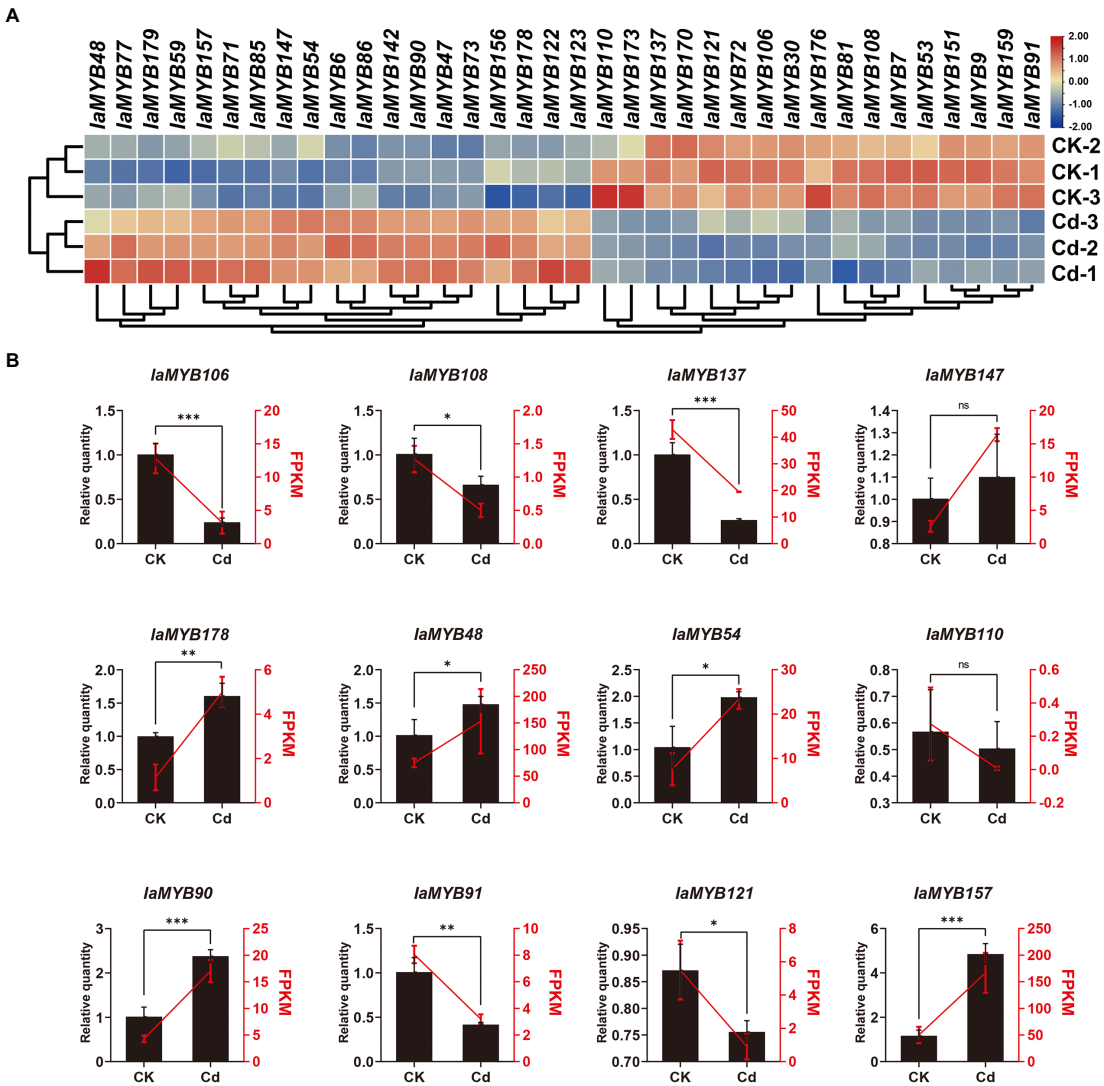
The expression patterns of the 36 DEGs. **(A)** Heatmap showing expression patterns of the 36 DEGs. **(B)** The expression of the 12 randomly selected DEGs validated by qRT-PCR. The CK represents the root samples in the control group, whereas the Cd represents the root samples in the Cd-treated group. Left Y-axis and black histograms indicate the relative expression levels quantified by qRT-PCR. The right Y-axis and red lines indicate FPKM values obtained by RNA-seq. The statistical analysis was performed using the student’s *t*-test. Data shows mean ± SD of three replicates (ns: no significance, ^*^p < 0.05, ^**^p < 0.01, and ^***^p < 0.01).

To illustrate the expression patterns of *IaMYB* genes in response to Cd stress, we quantified the transcript abundances of *IaMYB* genes by calculating the value of FPKM (fragments per kilobase per million mapped reads) of each gene based on the RNA-seq data (Supplementary Table S6). As shown in Figure 7A, a total of 36 differentially expressed genes (DEGs) were identified. Of these, more than 70% (26/36) of DEGs belonged to the 2R-type *IaMYB*. Moreover, 19 DEGs (including the seventeen 2R-type *IaMYBs* and two 1R-type *IaMYBs*) were up-regulated in the Cd-treated root, while 17 DEGs (including the nine 2R-type *IaMYBs* and eight 1R-type *IaMYBs*) were enhanced in the root of control group. According to the phylogenetic tree analysis, we found that most of DEGs were associated with defense (including drought, salt, hormone-mediated, light, wounding, and pathogen response), metabolism (including phenylpropanoid pathway, lignin biosynthesis), and development (including axillary meristem regulation, Lateral organ formation, and hypocotyl elongation) (Supplementary Table S7). The transcript abundances of four DEGs (*IaMYB47*, *IaMYB86*, *IaMYB142*, and *IaMYB147*) in the roots of the Cd-treated group were 5-fold higher than that of the control group, while five DEGs (*IaMYB7*, *IaMYB110*, *IaMYB121*, *IaMYB173*, and *IaMYB176*) were down-regulated more than five times in the root under Cd treatment.

To verify the accuracy and reliability of RNA-seq data, we randomly selected 12 DEGs for the relative quantification by using qRT-PCR. As shown in Figure 7B, the variation trend of FPKM obtained by RNA-seq datasets were consistent with the relative quantity of qRT-PCR, suggesting the RNA-seq data were accurate and reliable. Remarkably, according to the RNA-seq results together with the qRT-PCR analysis, we found that 2R-type *IaMYB157* was dramatically enhanced in the roots under Cd stress.

### Analysis of *cis*-acting regulatory elements of DEGs

**Figure 8:**
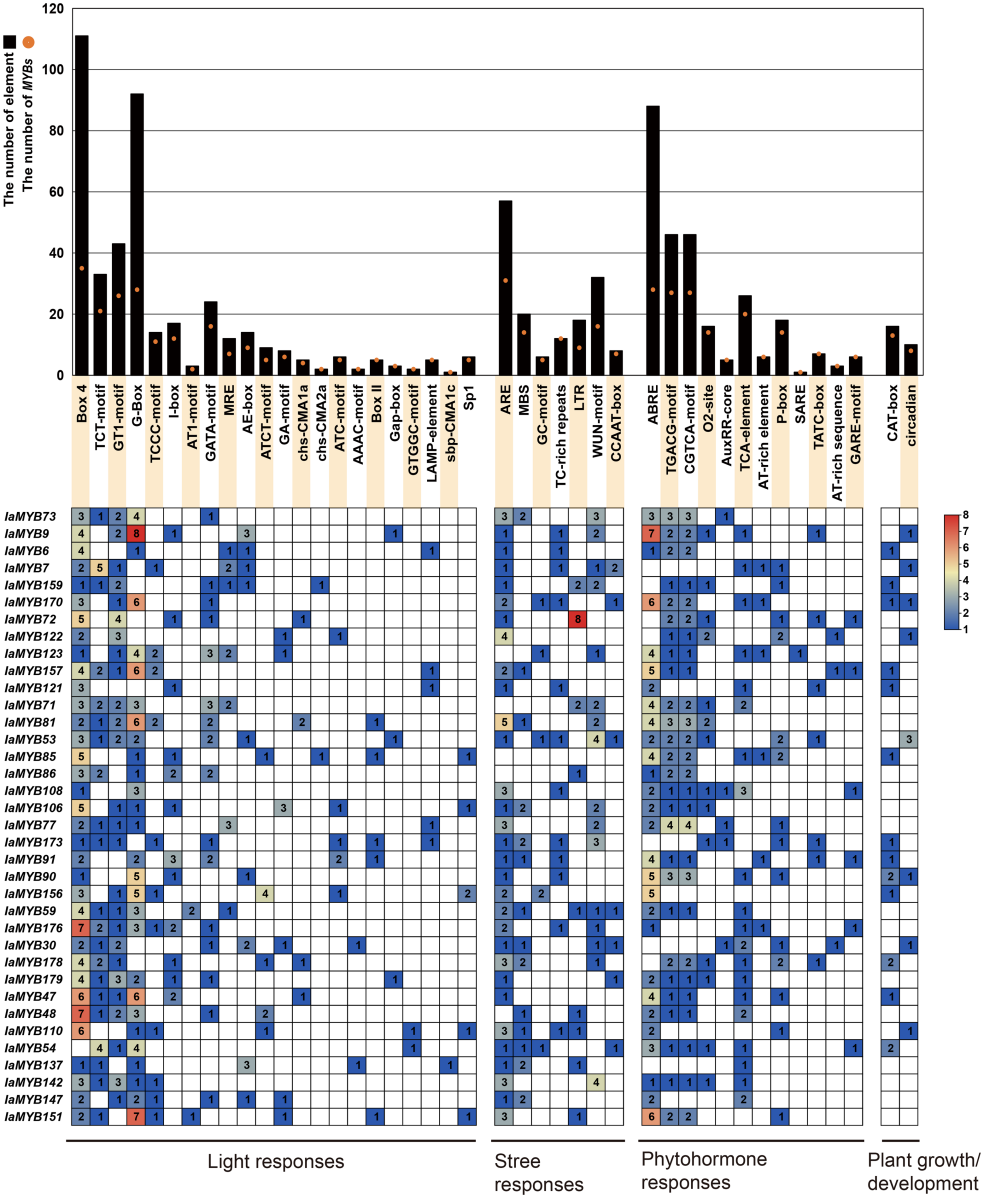
*Cis*-acting elements in the promoter region of the 36 DEGs. The histograms show the total number of the corresponding *cis*-acting elements in the promoter region of each DEG, whereas the orange dots show the total number of the DEGs containing the corresponding *cis*-acting elements. The heatmap was generated based on the number of the corresponding *cis*-acting elements in the promoter region of each DEG.

To explore the potential regulatory factors of 36 DEGs, the *cis*-acting regulatory elements of their promoter regions were analyzed by using Plant CARE online program. As shown in Figure 8 and Supplementary Table S8, a total of 43 types of *cis*-acting elements were found in 36 DEGs, which were classified into four major categories, including light-responsive elements, phytohormone-responsive elements, stress-responsive elements, and plant growth and development-related elements. Of these, the light-responsive elements are prevalent with 22 types (including Box-4, TCT-motif, and GT1-motif), followed by the phytohormone-responsive elements with 11 types (including the abscisic acid-responsive elements such as ABRE; the methyl jasmonic acid-responsive elements such as CGTCA-motif and TGACG-motif; the zeatin-responsive elements such as O2-site; the auxin-responsive elements such as AuxRR-core; the salicylic acid-responsive element such as TCA element; the gibberellin-responsive element such as P-box, TATC-box and GARE-motif; the other phytohormone-related element such as AT-rich and SARE), the stress-responsive elements with 7 types (including the anaerobic responsive elements such as ARE; the drought-responsive element such as MBS; the cold-responsive elements such as LTR; the wound-responsive element such as WUN-motif). While, only two types of the plant growth and development-related elements were detected, namely CAT-box and circadian. Moreover, *IaMYB9* had the largest number of light-responsive elements and phytohormone responsive elements, and *IaMYB72* had the most stress-responsive elements. *IaMYB53* and *IaMYB90* had the most plant growth and development-related elements. Remarkably, the 2R-type *IaMYB157* varied greatly under Cd stress, which harbored the abundant light-responsive element G-box and ABA-responsive element ABRE.

## Discussion

Myeloblastosis TFs are also known as trans-acting elements, which play a crucial role in regulating plant growth and development, biotic and abiotic stress tolerance, and biosynthesis of secondary metabolites. The release of a great deal of plant genome data provides important sequence resources for the genome-wide identification of the *MYB* genes family in plants. At present, the different numbers of *MYB* genes have been validated in several plant species with various genome sizes, including *A. thaliana* (*MYB* genes: 197; genome size: 125 Mb) (Stracke et al., 2001; Chen et al., 2006), *O. sativa* (*MYB* genes: 155; genome size: 466 Mb) (Katiyar et al., 2012) *Solanum lycopersicum* (*MYB* genes: 127; genome size: 828.349 Mb) (Li et al., 2016), *Solanum tuberosum* (*MYB* genes: 217; genome size: 840 Mb) (Li et al., 2021) *Morella rubra* (*MYB* genes: 174; genome size: 313 Mb) (Cao et al., 2021), and *Pyrus bretschneideri* (*MYB* genes: 129; genome size: 512 Mb) (Cao et al., 2016). In this study, a total of 183 *MYB* genes in 550.05 Mb genome of *I. aquatica* were identified. These results indicated that the number of *MYB* genes in different plant species were divergent, which were not proportional to the genome size of plant species.

Generally, MYB TFs were classified into four different types namely 1R-type MYB, 2R-type MYB, 3R-type MYB, and 4R-type MYB based on the arrangement of 1–4 incomplete repeats in their DBDs [18]. Among these, the 2R-type *MYB* genes are predominant in plants, which are probably derived from an ancestral 3R-type *MYB* gene through loss of the imperfect MYB repeat R1 (Jiang et al., 2004; Katiyar et al., 2012). In conformity with these findings, the 2R-type *IaMYB* contained the highest number of *IaMYB* genes, with 61.2% (112/183) of the total *MYB* genes in *I. aquatica*. The 4R-type *MYB* gene is also known as “Atypical *MYB* gene,” which was detected in diverse plant species. For instance, one 4R-type *MYB* genes in *A. thaliana* (Katiyar et al., 2012), two in *Gossypium hirsutum* (Salih et al., 2016), and four in *Brassica rapa* (Saha et al., 2016). However, there was no 4R-type *MYB* gene detected in some plants, including rice (Katiyar et al., 2012), sesame (Mmadi et al., 2017), and pepper (Arce-Rodriguez et al., 2021). In contrast to the 2R-type *IaMYBs*, the 4R-type *IaMYBs* constitute the smallest subfamily of MYB TF superfamily in *I. aquatica*, which contained only one gene. In addition, a *MYB* gene with five Rs was identified in *Arabidopsis* (Katiyar et al., 2012) and pepper (Arce-Rodriguez et al., 2021). While, we did not detect any 5R-type *MYB* gene in *I. aquatica*. The *MYB* gene containing only one *MYB* repeat or two separated *MYB* repeats are defined as *MYB*-related gene or 1R-type *MYB* (Jiang et al., 2004; Islam et al., 2021). 1R-type *IaMYB* constituted the second largest subfamily of MYB TF superfamily in *I. aquatica*. This finding is inconsistent with previous reports in sesame and chickpea where the 1R-type *MYB* was the largest subfamily of *MYB* genes family (Ramalingam et al., 2015; Mmadi et al., 2017). Additionally, some plants have more 3R-type *MYB* genes, while some plants have fewer (Qing et al., 2019). Overall, these findings indicate that the expansion level of different type of *MYB* genes varied in diverse plant species, which may be related to their distinct evolutionary strategies. The versatile functions of most MYB TFs can be comprehensively predicted by performing systematic evolutionary analysis (Wang et al., 2021b). Here, the evolutionary analysis is consistent with the previous report on *A. thaliana*. Most of IaMYB proteins were clustered together with well-defined AtMYB proteins, providing an excellent reference to predict the biological functions of *IaMYB* genes. Whereas, ten AtMYB proteins did not fit into any subgroups of IaMYB proteins, indicating the proteins with similar function might be lost in the genome of *I. aquatica* during its evolutionary process.

The analysis of physicochemical properties are helpful for deciphering the functional properties of proteins. The theoretical isoelectric point (pI) of a protein refers to the pH value at which the total charge carried by the protein is zero, which is a vital parameter for protein purification. Here, we found that pI values of IaMYB proteins were similar to those of MYB proteins in previous reports, suggesting the acidic (pI > 7.0) or basic (pI < 7.0) nature of IaMYBs (Halligan, 2009; Mohanta et al., 2019; Qing et al., 2019). The aliphatic index (Ai) reflects the thermostability of proteins. The protein with Ai value greater than 71 is considered thermostable (Hoda et al., 2021). Here, we found that 57 of 183 IaMYB TFs have a value of Ai greater than 71, indicating a thermostable nature of those IaMYBs. The instability index (Ii) reflects the stability of protein, and protein with Ii value less than 40 is considered stable. Consistent with previous reports, most of IaMYBs (171/183) were considered unstable (Li et al., 2022). The grand average of hydropathicity (GRAVY) reflects the hydrophobic or hydrophilic character of proteins. Proteins are referred to as hydrophobic if their GRAVY value is greater than zero, while hydrophilic if it is less than zero. In this study, we found that all IaMYB proteins had negative GRAVY values, suggesting those protein are soluble, an important nature that is necessary for TFs (Katiyar et al., 2012). As TFs, MYB proteins are mostly localized in the nucleus, where they play a crucial role in regulating downstream gene expression. Nevertheless, some MYB proteins were localized in the cytoplasm, mitochondria, or endoplasmic reticulum, and their roles in these organelles need to be further elucidated (Avila et al., 1993; Zhang et al., 2021; Sabir et al., 2022). Our results showed that all IaMYB proteins were localized in the nucleus. These findings implied that the *MYB* genes may performs their versatile functions in different organelles of plants. As mentioned above (Figure 4), *IaMYB* genes in the identical subgroup generally possessed similar motif compositions, which may share a similar function (Wang et al., 2021a). Remarkably, some *IaMYB* genes contained specific motif, suggesting these motifs may impart specific biological functions to those *IaMYB* genes. Understanding the exon-intron organizations within gene families allows us to obtain more clues about their evolutionary trajectory. Although the gene structures of each subgroup exist differences, most of *IaMYB* genes clustered together share similar exon-intron structural patterns, implying pivotal roles of these features in their evolutionary process and functional divergence. As reported, the Trp residues within MYB DBDs play a key role in DNA-binding specificity (Zhang et al., 2021). Here, we found that evolutionarily conserved Trp residues are regularly distributed between variable interval sequences within R2 and R3 MYB repeats of the 2R-type IaMYB, which is in line with previous studies. Noteworthy, the first Trp residue within R3 repeat was replaced by other amino acids, which may affect the sequence-specific binding of the MYB TFs.

The gene duplication events are the driving force for the expansion of gene families (Wang et al., 2012). Our results demonstrated that the DSD and WGD modes play key roles in the expansion of *IaMYB* gene families, which is similar to previous reports in different plants (Cannon et al., 2004; Liu et al., 2014; Sun et al., 2019). Furthermore, the analysis of Ka/Ks ratio showed that *IaMYB* duplicated gene pairs endured purifying selection, suggesting highly conserved evolution of *IaMYB* genes. The analysis of syntenic relationships between *IaMYBs* and three representative plant species including *I. batatas* (belongs to the family Convolvulaceae, dicot), *A. thaliana* (belongs to the family Brassicaceae, dicot), and *O. sativa* (belongs to the family Poaceae, monocot) confirmed that the same type of angiosperms from the identical family have closer evolutionary distance.

Heavy metal contamination has become a critical environmental problem. The accumulation of heavy metals in plants not only hampers plant yield and productivity, but threatens human health throughout the food chain (Alam et al., 2022). Cd is one of the most toxic heavy metal contaminants due to its long biological half-life and non-biodegradability (Cirmi et al., 2021; Altaf et al., 2022). Previous studies revealed that MYB proteins play a vital role in plant response to heavy metal stress. For instance, R2R3 AtMYB49 can directly or indirectly regulate the expression of HIPP and IRT1 proteins to enhance plant Cd accumulation, which also participate in ABA-mediated repression of Cd accumulation in plants by interacting with ABI5 (Zhang et al., 2019). The *BnMYB2* gene from *Boehmeria niveaw* was greatly enhanced by Cd treatment, which has been further demonstrated to improve plant Cd tolerance (Zhu et al., 2020). The gene encoding the heavy metal transporter (natural resistance associated macrophage protein, NRAMP) harbors MYB binding sites in their promoter region, indicating MYB TF may modulate its expression and thereby affecting heavy metal uptake and transportation in plants (Tian et al., 2021). Like in *Arabidopsis*, approximately 20% (36/183) of *IaMYB* genes were dramatically changed when the *I. aquatica* seedlings were subjected to Cd treatment (Chen et al., 2006). Of these, more than 70% (26/36) of significantly altered genes belonged to the 2R-type *IaMYBs*, inferring their significant effect on plant response to Cd stress. Analysis of *cis*-acting elements within the promoter region of the gene helps us to better understand the plant stress response. Studies showed that the light-responsive element G-box had the role of stress-responsive element, and the ABA-responsive element ABRE act as an important regulator of ABA-mediated stress response in plants (Abdullah-Zawawi et al., 2021). Here, we found that the 2R-type *IaMYB157* with abundant G-box and ABRE elements in its promoter region exhibited very strong response to Cd stress, indicating the possible function of *IaMYB157* in plant response to Cd stress.

## Conclusion

In conclusion, 183 *MYB* genes in *I. aquatica* were identified at whole-genome level, which were classified into four types (including 66 1R-type *MYB*, 112 2R-type *MYB*, four 3R-type *MYB*, and one 4R-type *MYB*) and categorized into 23 subfamilies (G1-G23). Bioinformatics analysis was conducted including chromosome distribution, syntenic relationship, sequence features, gene duplication events, and selection pressure. Furthermore, RNA-seq and qRT-PCR results demonstrated that approximately 20% of *IaMYB* genes had a significant role in *I. aquatica* roots under Cd stress. Remarkably, the 2R-type *IaMYB157* with abundant light-responsive element G-box and ABA-responsive element ABRE in its promoter region exhibited very strong response to Cd stress. Our findings provide an important candidate *IaMYB* gene for further deciphering the molecular regulatory mechanism in plant with respect to Cd stress.

## Data availability statement

The datasets presented in this study can be found in online repositories. The names of the repository/repositories and accession number(s) can be found in the article/Supplementary material.

## Author contributions

WB and ZL: conceptualization. WB, ZL, and YZ: methodology. ZL and YZ: software, validation, and writing—original draft preparation. ZL, YZ, MA, YH, GZ, XL, JZ, WM, and ZW: investigation. WB: funding acquisition, writing—review and editing, and supervision. All authors have read and agreed to the published version of the manuscript.

## Funding

This research was funded by the Hainan Provincial Natural Science Foundation of China (320MS008 and 2019RC059) and the Initial Funds for the High-level Talents of Hainan University (KYQD(ZR)1935).

## Conflict of interest

The authors declare that the research was conducted in the absence of any commercial or financial relationships that could be construed as a potential conflict of interest.

## Publisher’s note

All claims expressed in this article are solely those of the authors and do not necessarily represent those of their affiliated organizations, or those of the publisher, the editors and the reviewers. Any product that may be evaluated in this article, or claim that may be made by its manufacturer, is not guaranteed or endorsed by the publisher.

## Footnotes

^1^ https://www.arabidopsis.org/index.jsp

^2^ http://pfam.xfam.org/search

^3^ https://www.ncbi.nlm.nih.gov/Structure/bwrpsb/bwrpsb.cgi

^4^ http://smart.embl-heidelberg.de/

^5^ http://pfam.xfam.org/search/sequence

^6^ http://web.expasy.org/protparam/

^7^ http://www.csbio.sjtu.edu.cn/bioinf/plant-multi/

^8^ https://www.evolgenius.info/evolview-v2/

^9^ https://meme-suite.org/meme/tools/meme

^10^ http://weblogo.berkeley.edu/

^11^ http://bioinformatics.psb.ugent.be/webtools/plantcare/html/
